# Percutaneous transhepatic metallic stent insertion for malignant afferent loop obstruction following pancreaticoduodenectomy: a case report

**DOI:** 10.1186/1752-1947-6-198

**Published:** 2012-07-16

**Authors:** Isamu Hosokawa, Atsushi Kato, Hiroaki Shimizu, Katsunori Furukawa, Masaru Miyazaki

**Affiliations:** 1 Department of General Surgery, Chiba University Graduate School of Medicine, 1-8-1 Inohana, Chuo-ku, Chiba, 260-8670, Japan

**Keywords:** Malignant afferent loop obstruction, Pancreaticoduodenectomy, Percutaneous transhepatic biliary drainage, Self-expanding metallic stent

## Abstract

**Introduction:**

Malignant afferent loop obstruction following pancreaticoduodenectomy is a rare complication and may be fatal if suppurative cholangitis or obstructive jaundice develops. Effective and safe therapeutic strategies for malignant afferent loop obstruction following pancreaticoduodenectomy are scarce at present.

**Case presentation:**

A 51-year-old Japanese man underwent pancreaticoduodenectomy for carcinoma of the papilla of Vater. Seven months postoperatively, he developed a high-grade fever, jaundice, and right upper abdominal pain. Abdominal contrast-enhanced computed tomography showed afferent loop obstruction and intrahepatic bile duct dilatation due to nodal recurrence. Percutaneous transhepatic biliary drainage was performed, and a self-expanding metallic stent (WallFlex™ duodenal stent) was placed across the stricture using the transhepatic route.

**Conclusions:**

There are surgical and nonsurgical treatments for malignant afferent loop obstruction following pancreaticoduodenectomy. Nonsurgical treatments include either an endoscopic or percutaneous approach to the afferent loop. Of these methods, percutaneous transhepatic insertion of a self-expanding metallic stent is the preferred treatment for malignant afferent loop obstruction following pancreaticoduodenectomy because it is more prompt and less invasive.

## Introduction

Afferent loop obstruction following pancreaticoduodenectomy (PD) is a rare complication. Most cases of afferent loop obstruction following PD are malignant and may be fatal if cholangitis or jaundice develops [1-4]. Malignant afferent loop obstruction following PD presents a particular therapeutic challenge because the postsurgical anatomy usually prevents an endoscopic approach [2,5]. However, appropriate therapeutic strategies for malignant afferent loop obstruction following PD are scarce at present. We recently experienced a case of malignant afferent loop obstruction following PD treated with percutaneous transhepatic insertion of a self-expanding metallic stent (WallFlex™ duodenal stent), and we herein present this case.

## Case presentation

**Figure 1:**
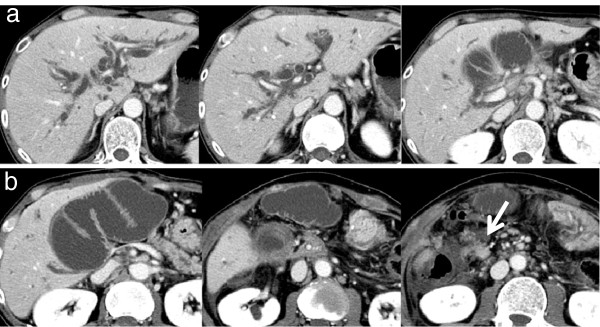
**Abdominal contrast-enhanced computed tomography on admission.** Abdominal contrast-enhanced computed tomography showed afferent loop obstruction and intrahepatic bile duct dilatation due to nodal recurrence (white arrow).

**Figure 2:**
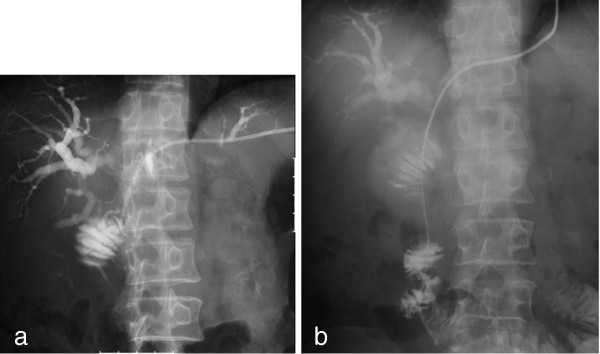
**Percutaneous transhepatic biliary drainage for treatment of malignant afferent loop obstruction.** (**a**) A 7 French pigtail catheter was inserted into the afferent loop from the tributary of segment 3. (**b**) The guide wire was advanced into the proximal aspect of the obstruction, and an 8 French endoprosthesis catheter was inserted.

**Figure 3:**
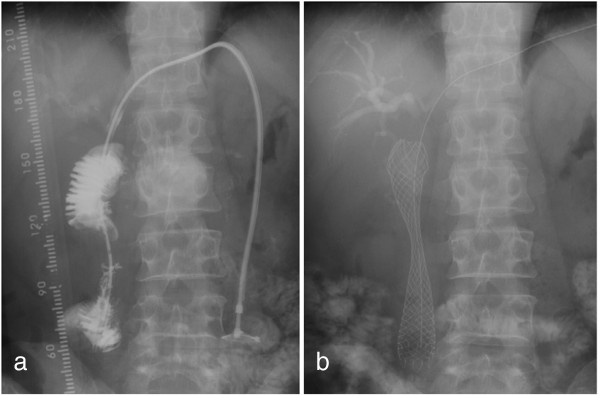
**Stent placement.** (**a**) Jejunography confirmed an approximately 4cm-long stricture of the afferent loop. (**b**) A 22mm × 12cm self-expanding metallic stent was placed across the stenosis via the transhepatic route.

A 51-year-old Japanese man underwent PD with Child reconstruction and Braun anastomosis for T4 N2 carcinoma of the papilla of Vater. Four of 34 resected lymph nodes were involved, but an R0 resection margin was achieved. Adjuvant chemotherapy with S-1 (tegafur-gimeracil-oteracil potassium) was performed after surgery. Seven months postoperatively, he developed a high-grade fever, jaundice, and right upper abdominal pain. The laboratory data on admission were as follows: white blood cell count, 9900/mm^3^; total bilirubin, 22.7mg/dL; aspartate aminotransferase, 199IU/L; and alanine aminotransferase, 178IU/L. The clinical diagnosis was suppurative cholangitis and obstructive jaundice. Abdominal enhanced computed tomography (CT) showed afferent loop obstruction and intrahepatic bile duct dilatation caused by nodal recurrence (Figure 1). To relieve jaundice and prevent cholangitis, ultrasound-guided percutaneous transhepatic biliary drainage was performed under local anesthesia. A 7 French pigtail catheter was inserted into the afferent loop from the tributary of segment three (Figure 2a). The guide wire was then advanced into the proximal aspect of the obstruction, and an 8 French straight endoprosthesis catheter was inserted (Figure 2b). The catheter was dilated from 8 to 12 French to deliver the self-expanding metallic stent. Jejunography confirmed an approximately 4 cm-long stricture of the afferent loop (Figure 3a), and a 22mm × 12cm self-expanding metallic stent (WallFlex™ duodenal stent, Boston Scientific) was placed across the stenosis via the transhepatic route (Figure 3b). On stent placement, we used the straight guide wire which was 0.035 inches in diameter and 450cm length. The next day, the expansion of the stent was good and balloon expansion was not needed. There were no procedural complications. His jaundice rapidly disappeared, and clinical conditions quickly improved. His hospital stay was 17 days. Stent patency was good, and cholangitis did not recur during five months of follow-up. He is currently receiving chemotherapy with gemcitabine as an outpatient.

## Discussion

Afferent loop obstruction is an unusual but serious complication following Billroth II gastrojejunostomy. Most cases are caused by benign obstruction of the afferent loop from an internal hernia, kinking at the anastomosis, or inflammation surrounding the anastomosis. Malignant occlusion is rare and generally caused by recurrent tumors or metastasis [3]. Obstruction of the afferent loop with ongoing accumulation of biliary, pancreatic, and intestinal secretions results in afferent bowel dilatation and subsequent biliary dilatation. Most of the clinical findings of afferent loop obstruction are relatively nonspecific and include abdominal pain, nausea and vomiting, fever, and obstructive jaundice. Almost all reported cases of afferent loop obstruction following PD involve malignant disease because of the high biological malignancy of the diseases for which PD is performed [2,6]. Afferent loop obstruction following PD easily results in cholangitis or jaundice because of the abolition of papillary functions [1-4]. Therefore, less invasive treatment for recurrent disease and immediate treatment for cholangitis or jaundice are required.

In the past, the incidence of afferent loop obstruction following PD was very rare. However, Pannala et al. [7] reported that the incidence of afferent loop syndrome following PD for pancreatic carcinoma was 13%, and survival of two years or longer was the only factor associated with the development of afferent limb syndrome. Recently, survival of pancreatic cancer has been extended due to the development of adjuvant chemotherapy. Therefore, the incidence of afferent loop obstruction following PD may be increased in the future.

We reported a successful outcome of percutaneous transhepatic insertion of a self-expanding metallic stent for malignant afferent loop obstruction following PD in a patient who was too unwell to undergo surgery and in whom the peroral approach could not be used because of altered upper gastrointestinal tract anatomy.

There are several treatments for malignant afferent loop obstruction following PD (Table 1). Surgery is effective but largely invasive [1]. Many patients with malignant upper gastrointestinal obstructions are not well enough to tolerate a surgical procedure [1,5,8]. Radical resection is usually impossible, and these patients are treated with jejunojejunostomy or Roux-en-Y bypass [8]. Surgical procedures are often difficult because of intra-abdominal adhesion and tumor infiltration and cause significant morbidity and mortality despite a short life expectancy [1]. Therefore, nonsurgical alternatives are clearly desirable [1].

**Table 1:** Surgical and nonsurgical treatments of malignant afferent loop obstruction

Surgical treatment	• Radical resection		
	• Surgical bypass (Jejunojejunostomy, Roux-en-Y bypass)•		
	• Jejunostomy		
Nonsurgical treatment	Peroral (Endoscopic) approach		· External drainage
			· Internal drainage
	Percutaneous	Direct route	· Balloon dilation
	approach	Transhepatic route	· Stent placement

An endoscopic approach to the afferent loop is less invasive, but is usually difficult because of postoperative anatomic changes [2,5]. Because of the high degree of afferent loop stenosis by the time patients develop symptoms such as jaundice, endoscopic treatment is mostly impossible [1]. The use of double-balloon endoscopy to approach the afferent loop in patients with surgically altered anatomy was recently shown to be safe and feasible [4,9]. A double-balloon endoscopic approach might be considered in selected patients with afferent loop obstruction.

Direct percutaneous puncture to the afferent loop is possible if the distended afferent loop underlies the anterior abdominal wall [1]. However, this procedure is complicated and there is a risk of bile leakage and subsequent peritonitis because the loop may decompress and collapse after puncture [2]. Therefore, it is necessary to fix the afferent loop to the anterior abdominal wall as well as perform gastrostomy to prevent peritoneal leaks [2].

The transhepatic route is effective and less invasive for initial treatment for cholangitis or jaundice. This route can also estimate the bilioenteric anastomosis patency [3,10]. However, biliary access can be challenging in patients with afferent loop symptoms without jaundice or dilation of intrahepatic biliary ducts.

In the present case, we chose the percutaneous transhepatic route for prompt treatment of severe cholangitis and jaundice. At the same time, we confirmed bilioenteric anastomosis patency. Because the stricture of the afferent loop was readily accessible by the transhepatic route, we naturally used this route for stent placement. We consider that this is a very simple and natural procedure to treat malignant afferent loop obstruction following PD with cholangitis or jaundice. There are several reports in which a 10mm biliary stent was used for the relief of malignant afferent loop obstruction. However, narrow biliary stents have a high risk of tumor ingrowth and occlusion. Moreover, biliary stents are more likely to migrate within the larger lumen of the small bowel. In this case, we chose a 22mm duodenal stent with a flare, which reduced the risk of migration and occlusion. Duodenal stents may be available with endoscopic (“through-the-scope”, or “TTS”) delivery systems, which are mostly 10 French. Thus, this stent can usually be advanced through the liver without too much difficulty.

Survival of patients who undergo metallic stent insertion for malignant afferent loop obstruction is likely to increase even further with improvements in palliative chemotherapy regimens [2]. Therefore, maintenance of stent patency is an important factor following stent insertion. The use of duodenal instead of biliary stents will delay reocclusion by tumor growth and should be considered to be the devices of choice.

## Conclusions

Several treatment modalities for afferent loop obstruction following PD exist, each with specific merits and drawbacks. Although treatment should be appropriate to individual cases, percutaneous transhepatic insertion of a self-expanding metallic stent is the preferable treatment for malignant afferent loop obstruction following PD because it is prompt and less invasive.

## Consent

Written informed consent was obtained from the patient for publication of this case report and any accompanying images. A copy of the written consent is available for review by the Editor-in-Chief of this journal.

## Competing interests

The authors declare that they have no competing interests.

## Authors’ contributions

IH was a major contributor to writing the manuscript. MM performed the initial operation. AK, KF and HS performed stent placement. KF has followed up this patient as an outpatient. All authors read and approved the final manuscript.
